# ACTA ORTOPÉDICA BRASILEIRA 15 ANOS DE JCR - JOURNAL CITATION REPORTS

**DOI:** 10.1590/1413-785220243204e050924p

**Published:** 2024-10-07

**Authors:** Olavo Pires de Camargo

**Affiliations:** 1. Universidade de Sao Paulo, Faculdade de Medicina, Hospital das Clinicas HC-FMUSP, Departamento de Ortopedia e Traumatologia, Sao Paulo, SP, Brazil.

 O *Journal Citation Reports* (JCR) é responsável pela atribuição do Fator de Impacto (FI) um método bibliométrico de avaliação de periódicos científicos, contabilizando as citações de cerca de 21.500 periódicos acadêmicos em mais de 250 disciplinas científicas. A entrada é concedida ao seleto grupo de periódicos que atendam aos altos padrões de qualidade, sendo 24 rigorosos critérios aplicados na avaliação. ^1^

 Apenas cerca de 15% dos periódicos passam pelo escrutínio da equipe editorial interna do Web of Science nesse padrão de alta qualidade. E a Acta Ortopédica Brasileira é o único periódico nacional de sua área a agregar a coleção JCR há 15 anos. ^1^

 Por quase meio século, as informações divulgadas no JCR são um importante recurso para identificar os principais periódicos confiáveis em suas áreas, garantindo a credibilidade das informações e dados fornecidos para promover e apoiar os objetivos coletivos da comunidade de aderir às normas de integridade de pesquisa. ^1^

 Baseado nas citações recebidas pelo periódico, o FI é resultado da soma das citações no ano do cálculo, dividido pelo total de artigos publicados nos dois anos antecedentes. Por exemplo, se um periódico publicou um total de 100 e obteve 50 citações, o fator de impacto desse periódico é de 0,5. ^2^ , ^3^

 A Acta Ortopédica Brasileira apresenta FI 0.5, o aumento da citação é sempre um desafio para periódicos nacionais e internacionais. ^4^ Para valorizar essa colocação da Acta, mantida há anos com trabalho sério, diário e voluntário chamamos a todos para ajudar a desenhar uma curva crescente significativa em nossas métricas, aproveitando integralmente seu potencial de citação, e levando a Acta Ortopédica Brasileira ao patamar merecido. O reflexo das ações propostas pode ser futuro, porém acreditamos que será estrutural à medida que for alcançado. 

Ressaltamos algumas iniciativas, considerando os princípios éticos das publicações científicas:

Vamos citar a Acta! Editores e avaliadores, como autores em outros periódicos podem e devem citar a Acta em seus artigos.

Editores e revisores da Acta Ortopédica Brasileira: “como revisores de outros periódicos podem sugerir a inclusão de referências (pertinentes ao tema) de artigos publicados na Acta nos artigos que estão avaliando para serem aceitos em outros periódicos.”

Conscientização junto aos departamentos, serviços, grupos e pós graduação para inclusão da Acta como literatura recomendada, adicionando nas referências dos artigos publicados em outros periódicos nacionais e internacionais.

Os artigos publicados na Acta Ortopédica Brasileira podem ser encontrados em importantes bases de dados, seu conteúdo é altamente indexável, pois é finalizado em “XML”, formato de programação mais atual e efetivo para conexão e visibilidade na rede. A busca por palavras-chaves para acesso a artigos do periódico pode ser realizada, por exemplo no PuMed, Scielo e no Google Scholar.

Abraço a todos,

Prof. Olavo Pires de Camargo

Editor Chefe

## References

[1] Clarivate lança Journal Citation Reports 2023 (2023). Agência de Bibliotecas e Coleções Digitais.

[2] Fator de Impacto (2021). PUC-Campinas.

[3] CAPES (2017). Tutorial de acesso de periódicos.

[4] Ruiz M. A., Greco O. T., Braile D. M. (2009). Fator de impacto: importância e influência no meio editorial, acadêmico e científico. Rev Bras Hematol Hemoter.

